# Supplementary material for: Lobelia chinensis Extract and Its Active Compound, Diosmetin, Improve Atopic Dermatitis by Reinforcing Skin Barrier Function through SPINK5/LEKTI Regulation
Source: Int J Mol Sci. 2022 Aug 4;23(15):8687. doi: 10.3390/ijms23158687 (PMC9369373; doi:10.3390/ijms23158687)
Supplement: Supplementary file 1 [file ijms-23-08687-s001.zip › ijms-1764793-supplementary.pdf]

Supplementary data

Lobelia chinensis extract and its active compound, diosmetin, improve atopic dermatitis by reinforcing skin barrier function through SPINK5/LEKTI regulation

No-June Park[1,2,†], Beom-Geun Jo[3,†], Sim-Kyu Bong[1], Sang-a Park[1], Sullim Lee[4], Yong Kee Kim[5], Min Hye Yang[3,*], Su-Nam Kim[1,2,*]

[1]Natural Products Research Institute, Korea Institute of Science and Technology, 679 Saimdang-ro, Gangneung, Gangwon-do 25451, Republic of Korea.

[2]Division of Bio-Medical Science and Technology, KIST School, University of Science and Technology, Seoul, 02792, Republic of Korea

[3]College of Pharmacy, Pusan National University, Busan 46241, Republic of Korea

[4]Department of Life Science, College of Bio-Nano Technology, Gachon University, Seongnam 13120, Republic of Korea

[5]College of Pharmacy, Sookmyung Women's University, Seoul 04310, Republic of Korea

[†]These authors contributed equally to this work.

***Corresponding author:**

Min Hye Yang,Ph.D., College of Pharmacy, Pusan National University, 2 Busandaehak-ro 63beon-gil, Busan 46241, Republic of Korea, Tel:+82-51-513-6754, Fax:+82-51-513-6754, E-mail:mhyang@pusan.ac.kr

Su-Nam Kim,Ph.D., Natural Products Research Institute, Korea Institute of Science and Technology, 679 Saimdang-ro, Gangneung, Gangwon-do 25451, Republic of Korea, Tel: +82-33-650-3503, Fax: +82-33-650-3529, E-mail: snkim@kist.re.kr

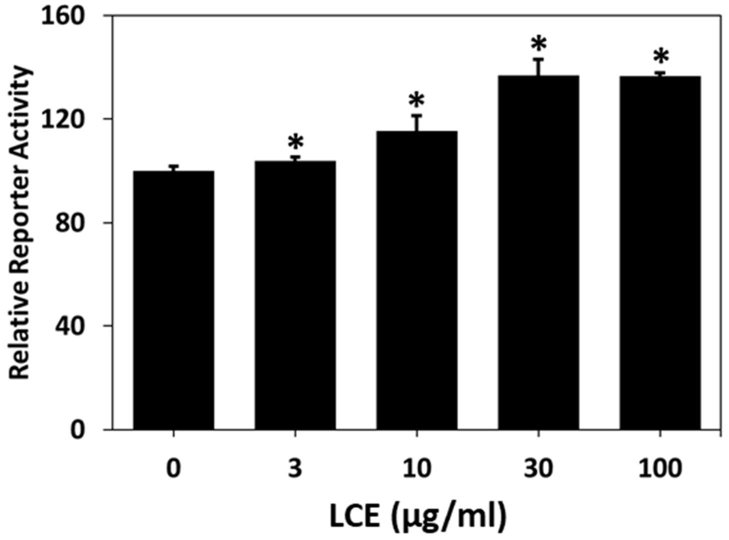

Supplementary Figure S1. Regulatory effect on SPINK5 of LCE. Relative reporter activity on SPINK5 gene expression of LCE was measured in CV-1 cells cotransfected the vector containing a SPINK5 promoter and vector containing reference universal promoter (SV40). Results are expressed as the mean ± SD of three independent experiments. *$p < 0.05$ vs. CON (control group). LCE: Lobelia chinensis extract, SPINK5: serine protease inhibitor kazal type-5.

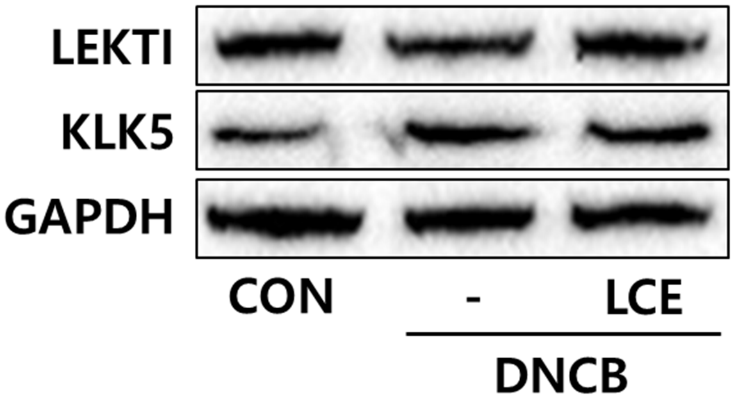

Supplementary Figure S2. Regulatory effect of LCE on LEKTI and KLK5 in the DNCB-induced model. These are representative blots of 5 mice. LEKTI: lympho-epithelial kazal-type-related inhibitor, KLK5: kallicrein 5.

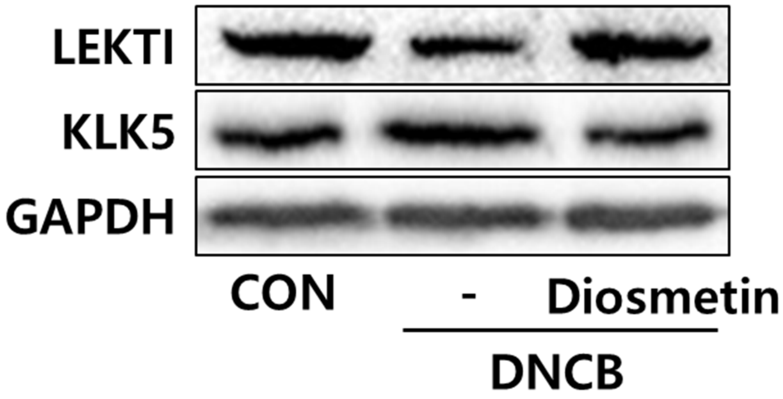

Supplementary Figure S3. Regulatory effects of diosmetin on LEKTI and KLK5 in the DNCB-induced model. These are representative blots of 5 mice. LEKTI: lympho-epithelial kazal-type-related inhibitor.
